# A Low Cost Real-Time Transient Recorder for High Voltage Systems

**DOI:** 10.3390/s23249769

**Published:** 2023-12-12

**Authors:** Selma Grebović, Nermin Oprašić, Ajdin Fejzić, Harun Kartal, Abdulah Akšamović, Samim Konjicija

**Affiliations:** Faculty of Electrical Engineering, University of Sarajevo, 71000 Sarajevo, Bosnia and Herzegovina; no14549@etf.unsa.ba (N.O.); afejzic2@etf.unsa.ba (A.F.); hkartal1@etf.unsa.ba (H.K.); aaksamovic@etf.unsa.ba (A.A.); skonjicija@etf.unsa.ba (S.K.)

**Keywords:** cloud storage, Industrial Internet of Things (IIoT), lightning strikes, remote monitoring system, Rogowski coil, transients in power system

## Abstract

Large-scale incorporation of new energy generation units based on renewable sources, such as wind and photovoltaic power, drastically alters the structure of the power system. Because of the intermittent nature of these sources, switching in grids (connection and disconnection) occurs much more frequently than with conventional sources. As a result, the power system will inevitably experience a large number of transients, which raises questions about the stability of the system and the quality of the electrical energy. Therefore, measuring various types of transients in power system is crucial for stability, power quality, fault analysis, protection design, and insulation design. Transient recorders that are currently used are generally expensive and only suitable for particular locations in power systems. The number of installed transient recorders is insufficient for a comprehensive analysis of problems that may occur. Hence, it is important to have inexpensive and efficient transient recorders that can be installed at multiple points in the power system on various types of objects. It is also essential to have a transient record database with open access, which can be used by researchers to develop new analysis techniques based on artificial intelligence. This paper proposes an inexpensive measurement and acquisition system designed to record transient phenomena on different objects within the power system. The system is designed to use autonomous power, a standardized data acquisition module, a low-budget system for transmitting recorded transient events to the server via mobile network, and a sensor system adapted to the object where transients are recorded. The proposed system is designed to be used for all types of objects in the power system where transients may occur, such as power lines, transmission towers, surge arresters, and transformers. All components of the system are described, and the system is tested under laboratory conditions. The modular nature of the system allows customization to the specifics of the location in power system by choosing appropriate components. The calibration method of the custom designed Rogowski coil is described. The cost analysis of the proposed system and power consumption analysis are performed. The results show that the system’s performance meets application requirements at a low cost.

## 1. Introduction

Disorders in the power system caused by faults, switching activities, lightning strikes, or load changes are known as transients. These phenomena can overload and damage electrical equipment [1,2]. Therefore, transient analysis [3,4] has become a fundamental methodology for understanding the sources of transients and selecting appropriate protection measures [5,6] against any type of stress. In this regard, surge arresters [7,8,9,10], various grounding techniques [9,11,12], grounding the neutral point through a shunt [13], or reactors are considered. The development of new techniques and software tools suitable for transient analysis of power systems is of great importance in the analysis and design of modern power systems. Sophisticated models, complex solution techniques, and powerful simulation tools must be validated and confirmed through measurement [14,15,16].

Therefore, the development of transient event measurement systems is an important and challenging task. Conventional current and voltage measurement transformers are designed to operate at the fundamental frequency of the power system and are not capable of measuring very fast transient events (above 1 MHz) [17]. Furthermore, they are only installed in substations and have a limited measurement range. On the other hand, very fast transients caused by lightning strikes require a minimum sampling frequency of 40 MHz [17]. To operate, maintain, and regulate the power system, it is necessary to preserve and analyze historical data collected in various operational modes. Having a large amount of information gathered from different sources is crucial for better power system analysis [18].

Hence, it is of great interest to install measuring equipment at various power system facilities and monitor transient events in the electrical power system. The design of measurement acquisition systems placed at these facilities is specific due to environmental conditions and the transient events that need to be recorded. Lightning Location Systems (LSS) are also used to analyze transient events caused by lightning strikes, which record the location and time of lightning strikes. These records are correlated with data from transient recorders with the aim of identifying the cause of the disturbance or fault, if one has occurred [15].

In paper [16], the use of optical current and voltage sensors for real-time recording of transient events in high-voltage substations is proposed. The aim is to expand the bandwidth of the sensors compared to conventional current and voltage measurement transformers. The paper mentions the use of a sampling frequency of up to 40 kHz with the possibility of extending it to 2 MHz. The proposed use of optical sensors would require replacing conventional current and voltage measurement transformers and would not be applicable for recording transients on transmission line towers or surge arresters. In [19], the authors describe a dedicated system with the option to record transients on a transformer by extending the existing transformer monitoring with the necessary components and a recorder for very fast transient events. In [13], the authors provide results of the application of a transient monitoring system on a transformer and a shunt resistor described in [19] in the context of the power system in Croatia. The system described in [13] uses voltage measurement transformers as sensors, while the expansion involves the installation of very fast data acquisition (DAQ). Such a system is usable only in substations and has a limited frequency spectrum. In [20], a developed transient recorder system is described, which uses fast wireless data transmission through wireless links to an internet node. It is suggested that multiple such measurement acquisition systems be installed in substations, time-synchronized via Global Positioning System (GPS), and transmit recorded events that can be correlated with other information at the receiving end. Data can be transmitted in either time or frequency domain formats. A 12-bit analogue to digital converter (ADC) with a sampling frequency of 100 Msps is used for sampling. To manage the distributed measurement system, an Field Programmable Gate Array (FPGA) device is used, providing the system with the necessary adaptability to the requirements of a specific application. The use of FPGA, due to the specific knowledge required for programming FPGA, may increase the system cost.

In paper [21], the authors presented a real-time transient recorder system installed on the telecommunication tower on Mount Lovćen (Montenegro) at an altitude of 1749 m. A current sensor was mounted at the top of the telecommunication tower to record lightning strikes on the tower. The system samples the lightning-induced signal in the current sensor with a sampling frequency of 7.8 MHz and 15-bit resolution using 4 channels. The recorded transient signals are transmitted via an internet connection to a server located at the Faculty of Electrical Engineering in Podgorica (Montenegro). The system is powered from the AC network at 230 V, and an industrial PC is used for managing measurements and data transmission. In [22], some results recorded on the system described in [22], are presented and discussed.

In [23], a system used for recording transients on a 220 kV and 500 kV power line is described. A specially developed optical sensor with a bandwidth of 5 Hz to 100 MHz was used as the sensor. Samples were collected using a fast 4-channel data acquisition system with a sampling frequency of 40 Msps and stored on a local data logger. In 2017, the system recorded 317 transient overvoltages caused by lightning strikes on the 220 kV power line.

Commercially available transient recorders are produced in two versions: the first one is used as a portable device for measuring transient events in power system facilities as needed [24,25], and the second one is integrated into a facility with a permanent function as a transient recorder [26]. Both types share the characteristic of being high-cost and utilizing existing current and voltage measurement transformers. In [27], the authors addressed the issue of unstable power supply leading to power outages in industrial facilities in Libya. To solve this problem, measurement systems with voltage monitoring and transient recording functions were installed at critical locations in substations. After analyzing recorded transients, it was found that transients led to incorrect operation of protective relays and system failures. Finally, a proposed solution involved replacement of some objects in the system, such as transformers, and adjusting the settings of protective devices to overcome the problem.

Transient recorders described in [13,18,19,22,23] are purposefully designed and deployed for use on a single object and a single location. They are typically installed in substations and use existing equipment for power supply, communication or as sensors. This applies even to commercially available transient recorders [24,25,26]. An exception is the concept proposed in [20], suggesting the use of a sensor network with wireless data transmission and time synchronization of data recorded at different locations through the GPS system. An example from [27] illustrates problems that can occur in complicated grids, which may result in the necessity of installing a large number of transient recorders. The system described in [22] represents an original idea for this work. The functionality of the system described in [22], successful lightning event recording, and longer reliable real-time operation encouraged the authors of this paper to elaborate on the system and propose its general use for transient recorders in the power system. Improvements introduced include open access to recorded events, autonomous power supply, low cost, easy installation, and usability for any type of object in the power system, tailored specifically to the application with sensor design such as the Rogowski coil.

Many authors [8,12,28,29] employ simulation models and tools to assess overvoltages caused by transients in power systems. This approach is justified, as it provides better insight into transient processes, and aids in correctly sizing equipment or evaluating equipment conditions. Even better results can be achieved with a sufficient number of measurements of these events to allow for higher-quality model estimation. In this regard, it is crucial to enable the installation of affordable and functional transient recording equipment on a larger scale, including locations beyond substations such as transmission line towers. When analyzing, estimating, and modeling transient signals, in addition to standard analytical methods, new techniques based on neural networks are also used [30,31,32,33,34].

The implementation of IIoT (Industrial Internet of Things) technologies within power systems is becoming an important infrastructure component that enables systematic data collection, analysis, and interpretation. This approach not only provides real-time information about the system’s state, but also allows for in-depth analysis of trends, anomaly detections, and anticipation of future needs and challenges. The affordability and cost-effectiveness of IIoT-based measurement systems is a determining factor for their widespread adoption, as it enables the installation of a larger number of devices throughout the power system. This, in turn, facilitates the collection of big data sets and allows a more comprehensive and detailed understanding of the system’s behavior. The development of affordable IIoT-based measurement systems is also a pivotal step towards creating smart power grids that are not only technologically advanced, but also economically feasible.

The motivation behind this research stems from the need to address the limitations of existing transient recording systems in power systems. Conventional measurement transformers struggle with very fast transients, hindering real-time analysis of crucial events. The integration of neural networks presents an opportunity to enhance signal analysis, but it requires a comprehensive database for effective training. The motivation also lies in providing a cost-effective, accessible, and versatile transient recording solution, expanding deployment possibilities beyond substations. Furthermore, the desire to contribute to the development of a more comprehensive understanding of transient events in power systems motivates the exploration of diverse deployment locations. In summary, the motivation is rooted in addressing technological gaps, improving analysis capabilities, and making transient recording more accessible, cost-effective, and applicable across various settings in the realm of power systems.

This paper describes and tests, under laboratory conditions, a transient recorder system with the following characteristics: it is based on IIoT with affordable components that meet the necessary sensor bandwidth, sampling rate, number of bits, and memory capacity requirements. The system utilizes inexpensive and accessible components for transmitting recorded events to Google Drive, providing access to a wide range of users and researchers. It has low energy consumption and uses an inexpensive self-contained power source, making it suitable for deployment both within and outside of substations.

Our contributions can be summarized as follows:

- Real-time very fast transient recording: the development of a transient recorder system with the capability to record very fast transients in real-time.
- Cost-effective and accessible solution: The proposal of an affordable transient recorder system, leveraging Industrial Internet of Things (IIoT) principles. This addresses the accessibility issue associated with many commercial solutions, making transient recording technology more widely available.
- Versatile installation options: Designing the system for installation both within and outside substations, including locations like transmission line towers. This extends the scope of transient recording.
- Practical value and importance of data collection: the research aims to contribute to the widespread implementation of affordable and functional transient recording equipment, fostering advancements in power system analysis and modeling.

The rest of this article is structured as follows. To demonstrate the significance and difficulty of developing a system that can record various transient types in power systems, in Section 2, overvoltages in power systems are briefly discussed, along with the role and significance of developed IoT solutions in industrial applications. The proposed system for measuring transients with elements of IIoT solutions is presented in Section 3. Section 4 reports the results of testing system in laboratory conditions. Section 5 includes an analysis of the costs of the developed system’s software and hardware components. System power usage and power supply strategies were also considered, and the paper is concluded in Section 6.

## 2. Overvoltages in Power Systems and Proposed Transient Recording System

Overvoltages in high voltage networks can be classified into two types: external overvoltages and internal overvoltages. External overvoltages are caused by lightning discharges into the elements of the power system or in its immediate vicinity. The value of lightning overvoltages depends on the energy of lightning discharge, but they are limited to lower values by applying appropriate protection methods. Internal overvoltages are caused by disturbances in the system itself. According to their impact on the insulation or the protective device, voltage stresses are categorized in IEC 60071 [35] and IEEE Std. 1894 [17] based on the duration of a power-frequency voltage or the shape of an overvoltage. Internal overvoltages are classified as temporary overvoltages, slow front overvoltages, fast front overvoltages, very fast front overvoltages, and combined overvoltages [17,35].

Power system transients cover a wide spectrum of signals, which are events ranging from several nanoseconds to several milliseconds. Power system transient peak values also span a range of values and depend on the type of overvoltage and other power system factors. Therefore, when attempting to accurately characterize and digitally represent analog signals such as voltage and current, it is important to take care that the waveform is presented credibly with its all-important parameters such as peak value, rise time, duration, and polarity. To represent the analog signal, an instrument takes discrete snapshots of the analog signal and converts them to their approximate digital equivalent. Many transient events are missed or not accurately recorded due to instruments with poor sampling rates. A higher sampling rate means more accurate representation of the waveform, but at the same time, the maximum sampling rate of available instruments depends on: vertical resolution (in bits), number of measuring channels, and memory of instrument (in MB per channel) [25]. Therefore, for optimal operation of measuring system compromise between mentioned features of measuring instrument is needed, but in accordance to specificity of events that should be recorded.

Lightning is unpredictable phenomenon that has significant influence on power system operation. Lightning events are fast front transients with time to peak usually between 0.1 µs and 20 µs, and tail duration less than 300 µs; therefore, measuring of these events is challenging task. Additionally, that of measuring is challenging due to occurrence of different lightning types: negative or positive polarity, or even bipolar. Also, it is not uncommon that one lightning flash is composed from more lightning strokes and all of them can strike the same place on the earth. According to [36], the time between two components in a flash is 30 ms. This means that the same point in power system can be affected by a few lightning strokes in very short time (in the ms range). Lightning current parameters of interest for better lightning protection and better insulation coordination in the power system are peak value, front time, tail time, duration, polarity and multiplicity. These parameters can be extracted from the lightning current waveform, and for that reason, it is very important to have appropriate system for measuring fast front transients [23]. The developed system must be able to record the lightning current waveform without losing any data. It is not easy task, since the quality of recorded data depends on sampling rate and resolution of measuring instrument. Also, if the system can record lightning events, then it is capable of recording most other transients.

The systems used for recording transients in the power system are typically expensive or of limited performance, rendering them unsuitable for creating a broader database of registered events. They are usually associated with existing parts of the power system, such as current and voltage measuring transformers, fault recorders in protective devices, data registration and transmission systems like SCADA, and power supply systems. As a result, in real-world operations, there is an insufficient number of installed such devices, typically installed as experimental equipment. A much wider application of transient recorders requires a change in the concept of designing such systems. They should make use of the existing infrastructure for other purposes, such as the internet, wireless data transmission, and IIoT. Furthermore, in a component-based design, very fast general-purpose data acquisition (DAQ) systems should be chosen, as well as affordable, cost-effective microcomputer systems, autonomous power supply systems, and cost-effective, wide-bandwidth current and voltage measurement transformers.

**Figure 1:**
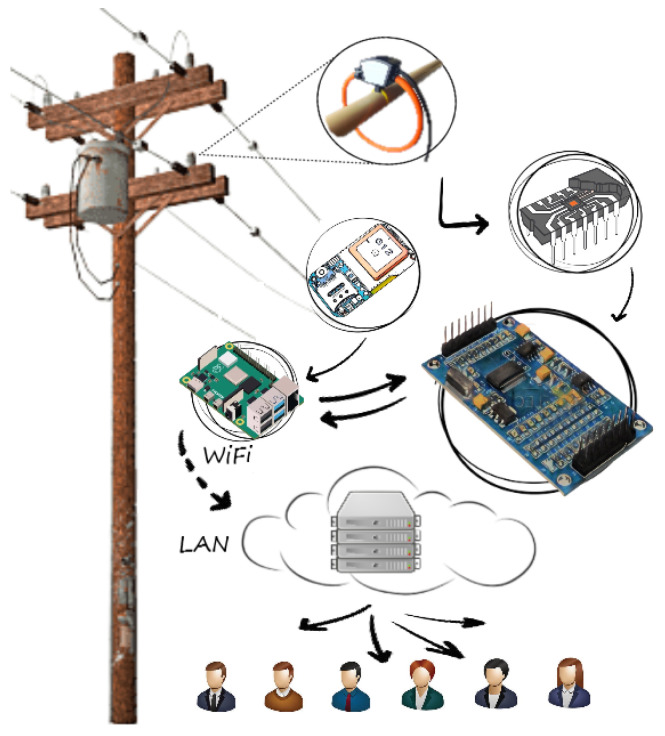
Concept of proposed transient recording system.

The concept of such a system is illustrated in Figure 1. As shown in Figure 1, the object on which transients will be measured can be any part of the power system, such as transformers, surge arresters, busbars, power lines, overhead line poles, grounding cables, neutral points, and so on. The system utilizes its own power supply, designed to meet the power consumption requirements of the transient recorder system, and is suitable for installation both inside and outside substations, such as overhead line poles, grounding points, surge arresters, disconnectors, and the like. Sensors are purposefully selected or manufactured to adapt to the object and the expected characteristics of the measured signal in terms of frequency characteristics, current and voltage amplitudes. The DAQ is chosen from the range of available general-purpose DAQ systems, so the number of channels, required vertical resolution, and the sampling rate match the application. Other system components (microcomputer, GPS, WiFi) are chosen in line with the available infrastructure in terms of internet access and the required software and hardware support for system function realization (management of data acquisition, filtering, storage, and data transmission).

## 3. Description of Proposed Transient Recording System

**Figure 2:**
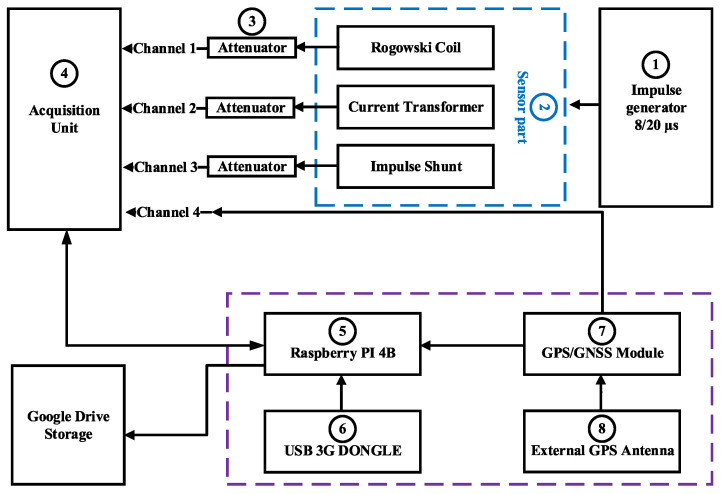
Block diagram of proposed transient recording system and testing in laboratory.

**Figure 3:**
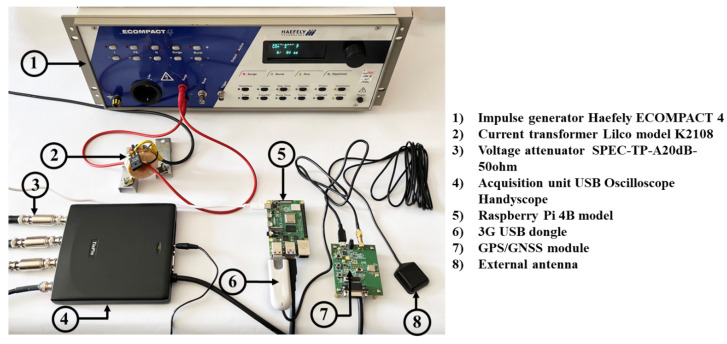
Testing setup that includes impulse generator and components of developed system.

A block diagram of the proposed transient recording system is presented in Figure 2. All components are labeled, and the corresponding experimental setup is shown in Figure 3. The system consists of following parts:

1. Impulse generator;
2. Sensor part (for testing purposes in this paper Rogowski coil, current transformer, and impulse shunt were used);
3. Voltage attenuator;
4. Data acquisition unit (DAQ);
5. Raspberry Pi;
6. USB 3G dongle;
7. Multi-GNSS module with embedded chip antenna;
8. External antenna;
9. Data storage part.

Lightning may create surge voltages that can harm the equipment in a few different ways. IEC 61000-4-5’s aim is to create a model to replicate these surges, so that it can be determined whether the equipment can withstand them [37,38]. The Ecompact 4 Haefely generator was chosen to produce current impulses, since it fulfills the requirements of both safety standards (IEC 61010-1) and standards for generating surges for testing procedures (IEC 61000-4-5) [39,40]. The main features of Ecompact 4 that are important for this work are given in Section 4.

### 3.1. Measuring Sensors

**Figure 4:**
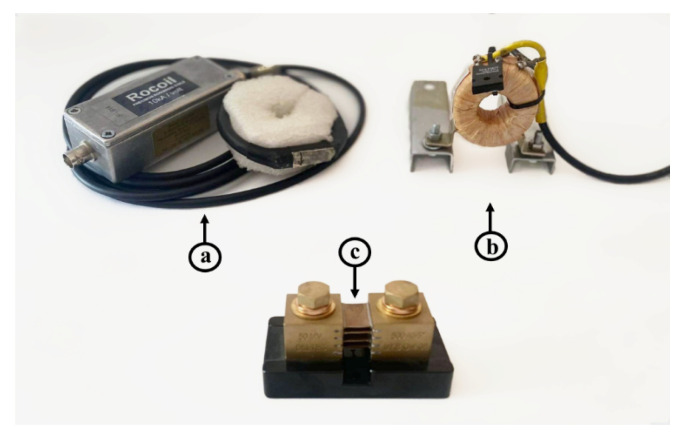
Measuring sensors: (**a**) Rogowski coil, (**b**) current transformer, and (**c**) impulse shunt.

Figure 4 shows three measuring sensors that are used for current shape recording:

(a) Rogowski coil;
(b) Current transformer;
(c) Impulse shunt.

Rogowski coil measurement is based on induced voltage in the torus of the coil. Induced voltage is proportional to the derivation of tested current, so that type of measurement requires an electronic integrator which reproduces same voltage shape as the shape of tested current. The main advantages include: large measuring currents range, linearity, flexibility, wide bandwidth greater than 10 MHz, and electrical isolation from the main circuit [41,42].

**Figure 5:**
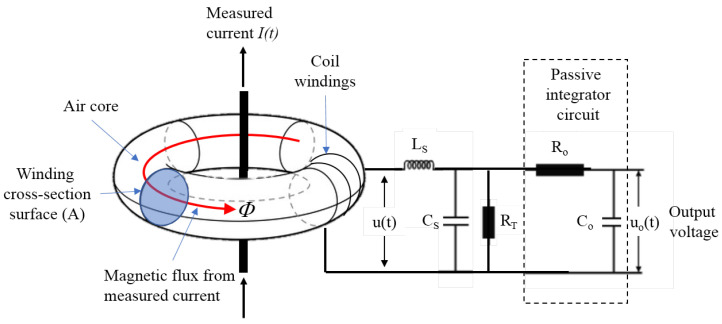
Rogowski coil with passive integrator.

For lightning strike current measurement, a typical Rogowski coil consists of a coil body and a passive integrator circuit, as illustrated in Figure 5. Characteristic parameters of the equivalent circuit of Rogowski coil transducer, shown in Figure 5, are: $L_{S}$ is the self-inductance of the coil, $C_{S}$ is stray capacitance, and $R_{T}$ is the terminating external resistor connected across the terminal of the coil. Integrator parameters are integrator resistance $R_{0}$ and capacitance $C_{0}$. Output voltage of the Rogowski coil u(t) is:

(1) $$u\left(t\right)=\frac{d\phi }{dt}=\oint \vec{B}d\vec{A}\;=\mu_{0}nA\frac{dI(t)}{dt}=M\frac{dI(t)}{dt}$$

where I(t) is measured current, $\mu_{0}$ is the permeability of free space, A is the winding cross-section area, *n* is the number of turns per length unit, and $\mu_{0}nA=M$ is the mutual inductance of the coil [41,43]. Output voltage $u_{0}\left(t\right)$ can be calculated as [41]:

(2) $$u_{0}\left(t\right)=\frac{1}{\tau }\int u\left(t\right)dt=\frac{M}{\tau }I\left(t\right)$$

where time constant $\tau =R_{0}C_{0}$. The ratio between the current being measured and the voltage output is defined as sensitivity of a Rogowski coil current transducer. Sensitivity in volts per ampere is [43]:

(3) $$\frac{u_{0}}{I}=\frac{M}{\tau }.$$

Sensitivity of the coil can be adjusted over wide range by adjusting number of windings and cross-section area, as well as integrator parameters [43].

The frequency bandwidth affects measurement accuracy of the peak value of a lightning current. Bandwidth BW of the Rogowski coil with integrator is given by [44]:

(4) $$BW=f_{h}-f_{l}=\frac{1}{2\pi }(\frac{1}{\sqrt{L_{S}C_{s}}}-\frac{1}{R_{0}C_{0}})$$

where $f_{h}$ is upper frequency and $f_{l}$ lower frequency limits. To achieve accurate measurement of lightning currents, the frequency bandwidth of the coil should be wide enough. As indicated by Equation (4), for maximal bandwidth, lower frequency $f_{l}$ should be as low as possible, while $f_{h}$ should be as high as possible. Therefore, $R_{0}C_{0}$ should be as high as possible, while the $L_{S}C_{S}$ as low as possible. Since $L_{S}$ and $C_{S}$ are parameters determined by the dimension and number of turns of the coil itself, geometrical parameters must be carefully investigated and selected to improve the design of Rogowski coil [44].

Tested current is proportional to the output voltage with ratio 10/1 kA/V. Design of the coil ensures that its easy to set up on phase conductor without the need of cutting the conductor. Output is not influenced significantly if the conductor is positioned ‘off-center’. The design also ensures that the influence from currents and magnetic fields external to the coil is minimal.

The Rogowski coil is suitable for use as a sensor for transient phenomena in the electrical power system [41,45,46]. This coil can be custom designed to meet specific requirements of the object in terms of physical dimensions, transient amplitude range, and frequency range of transient events. It is particularly well-suited for use on overhead transmission line towers or for mounting on existing installations such as surge arresters, protective ropes, etc.

**Figure 6:**
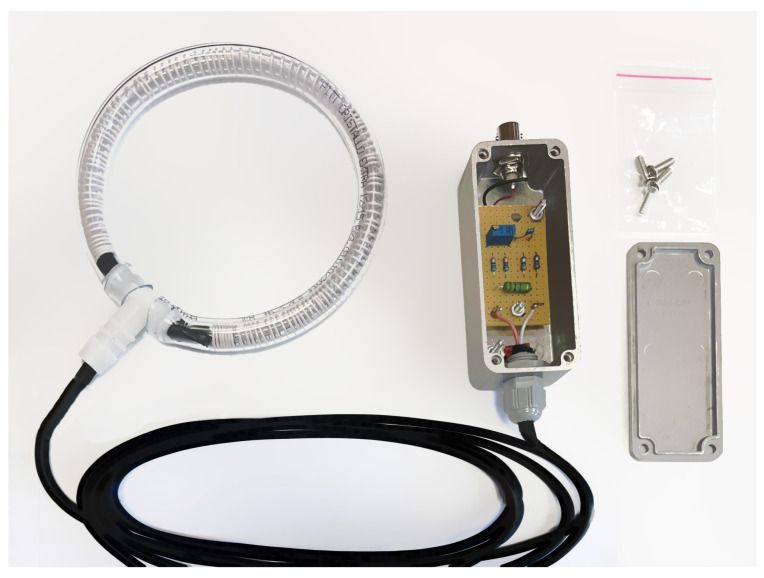
Custom designed Rogowski coil.

**Figure 7:**
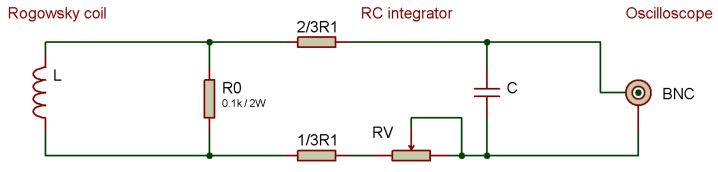
Schematic of Rogowski coil integrator circuit.

Figure 6 shows a custom designed Rogowski coil with a corresponding integrator. The flexible core allows for the coil to be placed around the object’s body (such as a conductor, busbar, overhead transmission tower, etc.) without the need for additional interventions on the object. The induced voltage in the coil is then fed into an oscilloscope according to the scheme depicted in Figure 7. The integrator is designed as a passive RC low-pass filter, with the cutoff frequency chosen in accordance with the required bandwidth. The choice of capacitance *C* and resistance *R* in the integrator can be adjusted to alter the transfer ratio, effectively allowing for calibration. For precise calibration, *R* is realized as a series connection of a fixed resistor $R_{1}$ and a variable resistor $R_{V}$, where $R_{V}$ is a multi-turn variable resistor with fine-tuning capability.

Current transformers are usually performed without a classical primary coil, but only with a conductor placed through; this is also the case with the specific model Lilco K2108. On the secondary side, there is a 50 Ω resistor on which the voltage is measured. Resistor voltage is proportional to the tested current on primary side with transfer ratio 1/0.25 A/V. The maximum current peak that can be measured with this transformer is 5000 A, after which the ferromagnetic core is saturated. Permanently RMS value of the current is 50 A [47]. The currents are also measured with a conventional laboratory measurement system. This system consists of an oscilloscope and impulse shunt shown in Figure 4c. Voltage is measured at low resistance and shunt has an approximate resistance of 0.0005 Ω. The current is obtained via this voltage drop.

### 3.2. Voltage Attenuator

An attenuator is a device that lowers the signal’s amplitude by a known amount and is used in a variety of applications. When a signal has to be weakened to safeguard measuring hardware or other electronics or to increase the range of power meters, voltage attenuators can be utilized. By reducing the signal level to an ideal range, voltage attenuators help avoid signal overload in receivers and detectors.

In order to protect acquisition unit and increase its range, voltage attenuator model SPEC-TP-A20 dB-50 ohm (see Figure 3, the device labeled with number 3) was used. The used voltage attenuator has the following basic characteristics: voltage ratio 10:1, midband accuracy ±1%, input and output resistance of 50 Ω, usable rise time 5 ns, frequency range: DC—3 GHz [48]. For example, to provide safe recording with an oscilloscope, the Lico transformer has to be adjusted to an appropriate level suitable for safe recording. If the attenuator’s transfer ratio is 10 V to 1 V, the system’s total ratio is 1 A to 0.025 V; for a maximum of 80 V at the oscilloscope input, the system can measure a current of 3200 A.

### 3.3. Data Acquisition Unit (DAQ)

USB oscilloscope Handyscope HS6-1000 (TiePie engineering, WL Sneek, Netherlands) was used to observe the waveforms of output signals. It has four channels with a maximum sampling rate of 1 Gsps (that is even suitable for recording very fast front transients) and a resolution of 8, 12, 14, or 16 bits. To determine the samples, the oscilloscope uses an internal A/D converter with a very precise clock to determines the moments at which sampling needs to be performed. The measuring range of input voltages is between ±200 mV and ±80 V, and the connection to the instrument is made via a cable [49]. The oscilloscope has the capability to adjust the recording of a transient event triggered by exceeding a specified voltage threshold. When a voltage with a magnitude greater than the trigger setting occurs, a sequence of input data are stored, where 20% (pre-trigger) represents the data before the trigger, and 80% is recorded after the trigger. The length of the recording can be selected by changing the sampling frequency.

Power and data transfer is provided via USB 3.0 connector; if the USB cannot deliver enough power, it is possible to power the instrument from an external power supply. The Handyscope HS6-1000 has a 3.5 mm power input located on the back of the instrument. The indication that the device is powered is made in the form of an LED indicator located on the top of the device. For data transfer interface USB 3.0 SuperSpeed (5 Gbit/s) is available.

Software support comes in the form of a program intended for use on Windows 7, 8, and 10 operating systems. For other operating systems, support is provided in the form of a “software development kit” that allows developing of one’s own software. Python and the software development kit were used to develop the embedded solution graphics application for this project.

### 3.4. Raspberry Pi

As a computing part of the system, the embedded board Raspberry Pi model 4B (small credit-card sized computer shown in Figure 3 and marked with number 5) was used. There are many developed industrial applications that are based on Raspberry Pi, such as: real time industrial energy analyzers, power consumption in smart homes, cloud-based light intensity monitoring systems, industrial Raspberry Pi, Programmable Logic Controllers (PLCs), etc. [50,51,52,53]. The advantage of this device is compact size, small weight, and low price.

Model 4B is equipped with an integrated 64-bit quad core ARM Cortex-A72 processor, with a speed that goes up to 1.5 GHz. The internal RAM is 4GB LPDDR4 RAM [54]. Storage for programs and data can be provided using a 16 GB micro-SD memory card or with NVME SSD, which can be used with the Raspberry Pi.

The device also supports WiFi, Ethernet and Bluetooth connectivity modes and an HDMI port for displaying image and sound on TVs or monitors. The device is powered via a micro-USB adapter at 5 V/3 A. It has 2 USB 2.0 and 2 USB 3.0 ports for connectivity. One USB 3.0 port was used for an acquisition unit for data transfer between the acquisition unit and Raspberry Pi, and the other was used for the 3G dongle, which provides an internet connection in areas where no other connection method is available. A USB 2.0 port was used for the GNSS module with an embedded chip antenna.

A free operating system Raspberry Pi OS based on Debian is installed, which is optimized for the Raspberry Pi hardware. Raspberry Pi runs embedded solution software developed exclusively for adjusting and controlling the lightning discharge measurement system.

### 3.5. Huawei E303 3G Modem Dongle

To provide internet connection in areas where no other connection method is available, a Huawei E303 USB 3G modem dongle (in Figure 3, marked with number 6) was used. This device connects via one of the USB ports and uses 3G mobile internet to transfer data. For that function, the device needs a standard Subscriber Identification Module (SIM) card that is inserted into the suitable SIM slot. The card must be able to use mobile internet. The device is unlocked for all domestic networks so that the user chooses the operator of their choice based on signal coverage, benefits and tariffs that he earns for the use of mobile data. The data transfer speed is limited to the speed of 3G mobile internet. For the purpose for which this device is used, where the data transfer rate is not crucial, the use of a 3G modem completely satisfies the desired needs.

### 3.6. Multi-GNSS Module with Embedded Chip Antenna

L96 (marked with number 7 in Figure 3) is a multi-GNSS receiver module with an embedded chip antenna. It supports GPS, GLONASS, Galileo, BeiDou, and QZSS navigation systems. Also, L96 supports multiple power saving modes to make the system work on the lowest power consumption. The maximum power requirement is 82.5 mW (25 mA and 3.3 V) provided through micro-USB port [55]. The embedded GNSS antenna enables customers to easily design smaller end devices without any performance loss. The purpose of the GNSS module in this system is to achieve precise time synchronization of system using satellite pulse per second (PPS) signal. PPS signal is an electrical signal that sharply rises or falls, and that accurately repeats once per second. Timing accuracy is equal or less than 10 ns. Additionally, an external antenna is supported for better usage of this module for indoor applications. An external antenna is also shown in Figure 3 and labeled with number 8.

### 3.7. Measurement Management and Data Storage

Before starting the measurement, it is necessary to set the measurement parameters. For that purpose, the embedded solution graphic software is developed, where the parameters are set by selecting the appropriate options in the drop-down menus. Data collecting is based on measuring a fixed number of points with a certain sampling frequency in the moment of discharge trigger. Data are firstly stored in the internal memory of the oscilloscope, and then transferred and stored on the computer memory via USB 3.0 connection. The only pause in measuring occurs during data transfer from the internal memory of the oscilloscope to the memory of the computer. After data transfer, the oscilloscope frees internal memory, and starts a new cycle of measuring. To speed up this process and reduce pause time, threading functions are used. Threading functions solve the problem of waiting for one task to be completed in order to start another. The threading option is also used for making copy of collected data to Google Drive. Before uploading, the data are converted to RAR format and, as such, are stored in Google Drive. This significantly saves drive memory, reduces mobile data traffic, and finish uploads faster. The upload process starts automatically at the user selected time once per day. This feature allows access to collected data from any computer with an internet connection. It also offers the option of an automatic power-on. The software was developed in Python.

## 4. System Performance Testing

The developed transient recorder was tested in laboratory conditions using the setup shown in Figure 3. Tests were designed to simulate large number of lightning strikes during long periods of time. For a continuous period of three months, the recorder system was repeatedly triggered by an impulse generator at random time intervals, and data were processed and recorded on cloud server. During that time, no problems with measuring equipment, data transmission, or recording were observed. Some examples of the recorded data are included in this paper. Other types of data (GPS data, etc.) recorded by this system are omitted from this work; nevertheless, it might be important to note that time is recorded in nanoseconds in order to obtain precise lightning event time. The following tests were performed:

- Recording of current shape of positive and negative polarity;
- Real-time data transfer;
- Automated power-on option.

**Figure 8:**
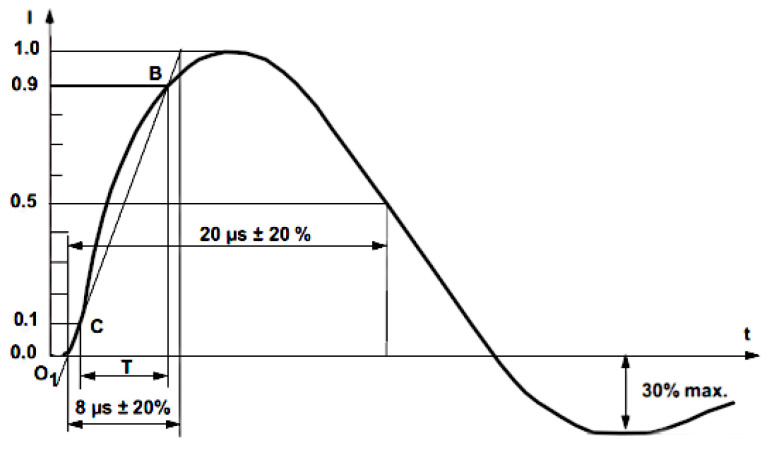
Current waveform 8/20 µs in short circuit.

The impulse generator Ecompact 4 created an 8/20 µs current waveform with various amplitudes to test the operation of the acquisition system and measurement sensors. The most important features of used generator are output current 0.1–2.1 kA with resolution of 0.1 kA, current rise time 8 µs ±20%, and current duration 20 µs ±20%. The voltage waveform produced by the generator is 1.2/50 µs in open mode and current waveform 8/20 µs in short circuit. Combination wave generators are the name for this sort of generator. Figure 8 shows the typical current waveform that was used for the experiments conducted in this study [56,57].

### 4.1. Measured Current Waveforms

**Figure 9:**
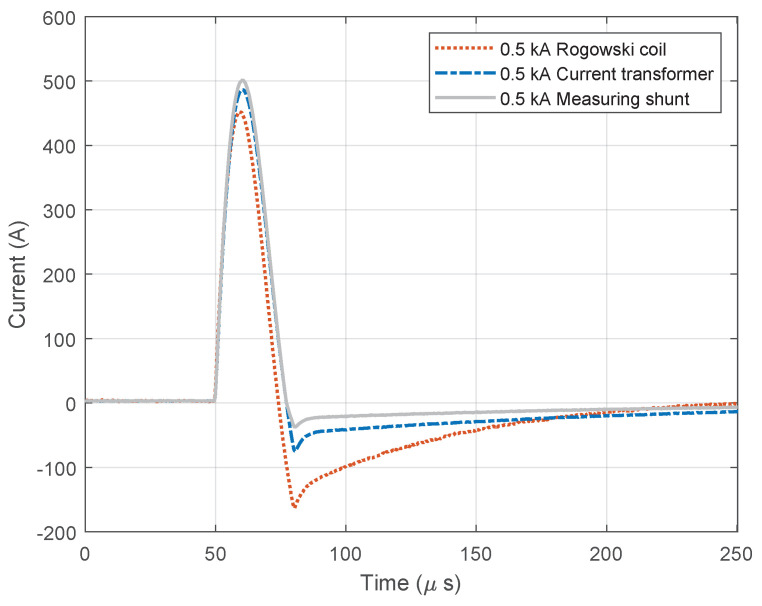
Positive current waveforms measured with different devices and for amplitude of 0.5 kA.

**Figure 10:**
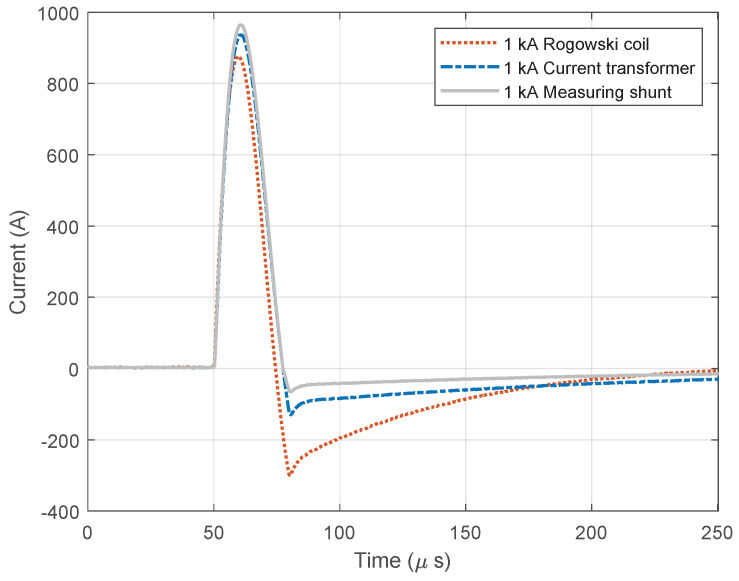
Positive current waveforms measured with different devices and for amplitude of 1.0 kA.

Figure 9 and Figure 10 show diagrams of the measurements performed by using three different measuring sensors (shown in Figure 4). The impulse generator current in each experiment (labeled “Measuring shunt” in figures) was measured as voltage drop on the low-resistance shunt resistor (shunt resistor parameters are detailed in Section 3.1). During measurement, all three different sensors were connected to their channels and measured the same signal source, ensuring that all three devices recorded the same signal. Devices were exposed to positively polarized current pulse with amplitudes of 500 A (Figure 9) and 1000 A (Figure 10). The oscilloscope sampling frequency during this test was set to 10 MHz. The total duration of the measurement was 250 µs, in which 50 µs (20%) refers to the pre-sample part, while the remaining 250 µs refers to the current pulse. The system recorded a current pulse of 500 A on the date 10 April 2023 at time 9:40:1.226894121, while current pulse of 1000 A was recorded at 9:45:2.865445223.

**Figure 11:**
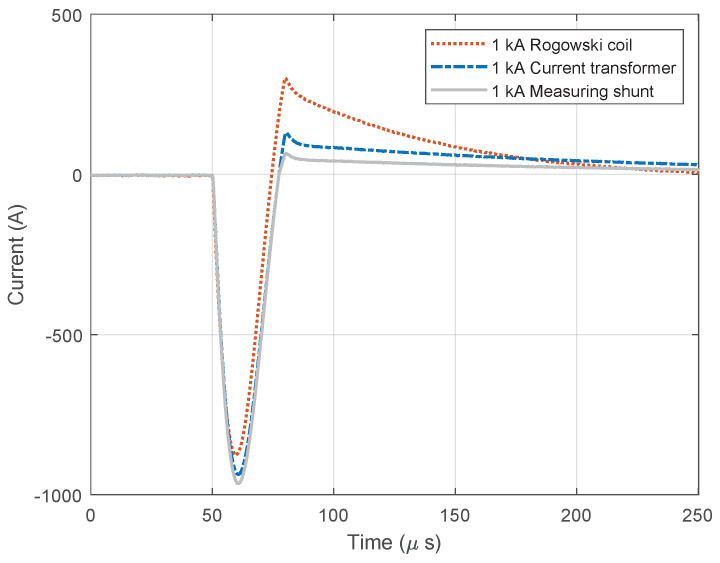
Negative current waveforms measured with different devices and for amplitude of 1.0 kA.

**Figure 12:**
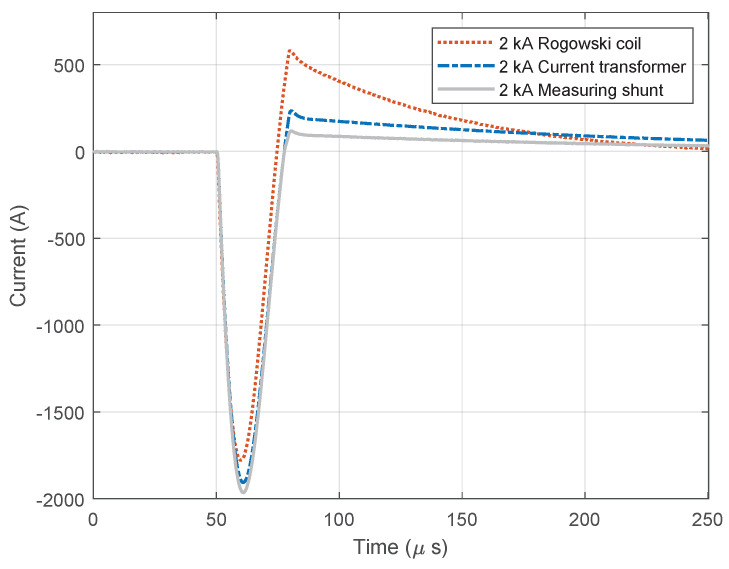
Negative current waveforms measured with different devices and for amplitude of 2.0 kA.

Figure 11 and Figure 12 show diagrams of negatively polarized current pulses with amplitudes of 1000 A (Figure 11) and 2000 A (Figure 12) applied to the devices. During this test, the oscilloscope’s sampling frequency setting was 10 MHz. The measurement was taking place for a total of 250 µs, of which 50 µs (20%) referred to the pre-sample part and the remaining 250 µs to the current pulse. The system recorded negative current pulse of 1000 A on the date 12 April 2023 at time 13:12:3.682422121, while negative current pulse of 2000 A was recorded at 13:22:16.269847632.

### 4.2. Rogowski Coil Calibration

**Figure 13:**
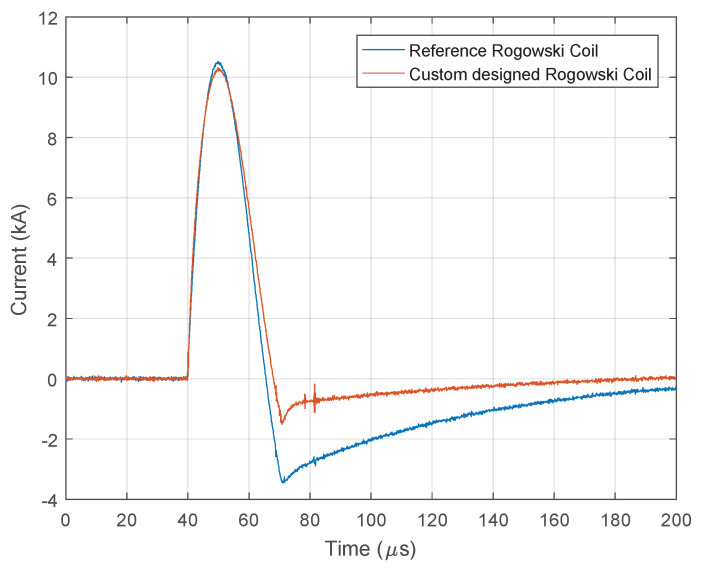
Custom designed Rogowski coil calibration.

The signals shown in Figure 9, Figure 10, Figure 11 and Figure 12 differ in the measured amplitudes of the current pulse depending on the sensor. In the real system, only one sensor will be used, which needs to be calibrated before use. Figure 13 illustrates a comparison of results for a test impulse between a custom-made Rogowski coil and a reference Rogowski coil calibrated in accordance with the procedure described in [56,58]. From Figure 13, it is evident that signal from the custom-made coil closely matches the signal from reference Rogowski coil. Only a slight delay in the signal’s falling edge and a slightly lower signal attenuation is observed. The diagram in Figure 13 is obtained based on the generated signal from the Ecompact 4 generator, which provides a maximum of 2.1 kA. In order to obtain higher current values on the sensor, the winding from the generator has encircled the receiving probe on the Rogowski coil multiple times (five times). This allowed for the registration of amplitudes up to 10 kA.

### 4.3. Electromagnetic Interference on Rogowski Coil

The measurement sensors will be positioned on locations where the normal presence of strong electromagnetic fields and electromagnetic interference (EMI) caused by transient processes is expected. The Rogowski coil is designed so that the induced voltage is proportional to the current flowing through the conductors encompassed by the coil [43]. The induced voltage at the ends of the Rogowski coil is proportional to the number of turns in the coil, as this voltage is obtained as the sum of voltages in all individual turns. In cases where the current conductor is located outside the coil, at half of the winding, the direction of the induced voltage is one way, and on the other half, it is the other way around. This directly follows from Equation (1), as at half of the winding, the scalar product between $\vec{B}$ and $d\vec{A}$ will be positive, and at the other half, it will have the same negative value. This will result in the cancellation of these voltages, and the resulting voltage will be zero.

The above holds true in the case of ideal geometry and symmetry. However, in real conditions and with real coils, these assumptions will not always be met. Therefore, we conducted experiments to determine if there is a significant impact of external influences on the measurement results.

**Figure 14:**
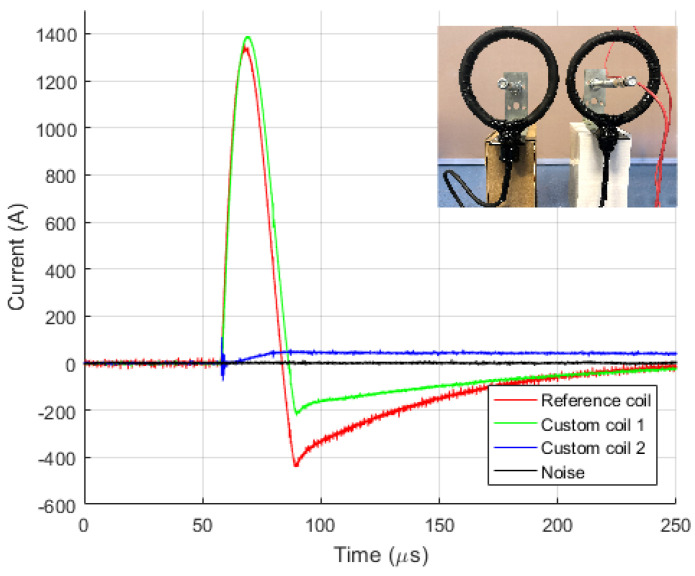
Measurement on Rogowski coil at distance 0.1 m from current carrying conductor.

**Figure 15:**
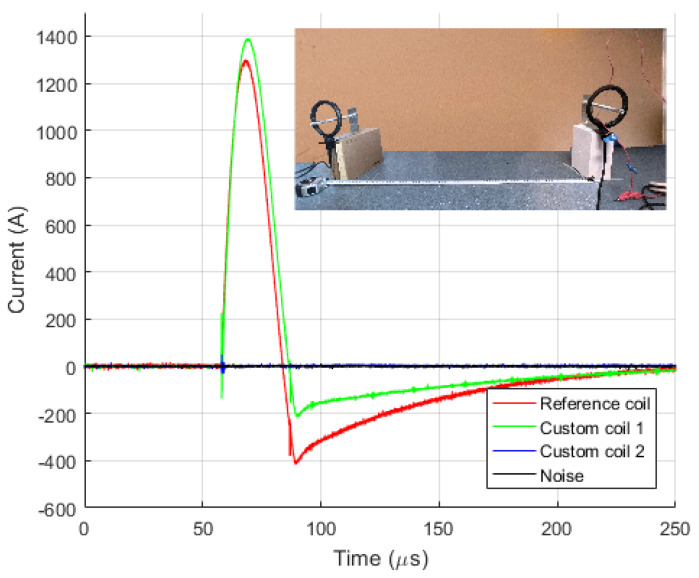
Measurement on Rogowski coil at distance 1 m from current carrying conductor.

To verify the extent of influence of physical realization and coil placement, relative to the current carrying conductor, to interference from sources not encompassed by the coil, two experiments have been conducted, and the results are shown in Figure 14 and Figure 15. In Figure 14, the coil not encompassing the conductor is positioned at a distance of 10 cm from the conductor, while in Figure 15, this distance is 1 m.

Four channels on the oscilloscope have been used for measurement. The first channel provides the reference coil signal (red color) and encompasses the conductor. The second channel provides the signal from the custom designed coil (green color) and encompasses the conductor. The third channel provides the signal from another custom designed coil (blue color) that does not encompass the conductor. The fourth channel measures noise, i.e., the open input of the oscilloscope (black color).

In Figure 14, the coil not encompassing the conductor registered the impact of EMI, reflected in a small offset in the measured signal with an amplitude of 3.2% of the value of measured current amplitude. In Figure 15, the signal from the coil not encompassing the conductor coincides with the noise signal. Based on the measured signals, it can be concluded that the impact of EMI on Rogowski coils is negligible. Still, it is recommended to place them at the maximum possible distance from potential EMI sources, following the prescribed minimum distances between equipment.

### 4.4. Real-Time Data Transfer

**Figure 16:**
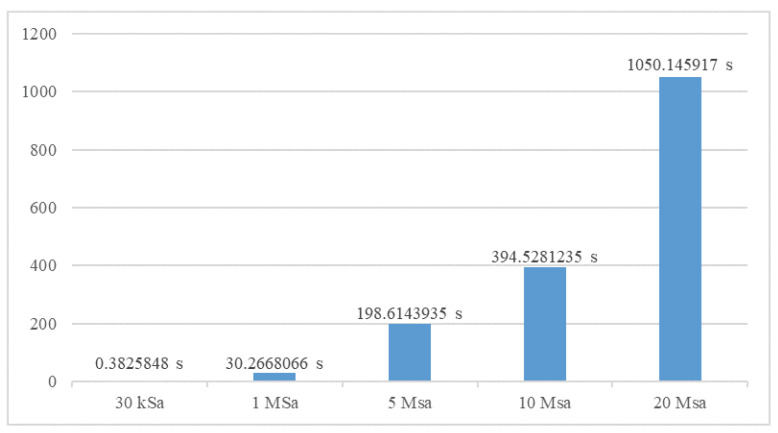
Pause time between measurements without threading functions.

**Figure 17:**
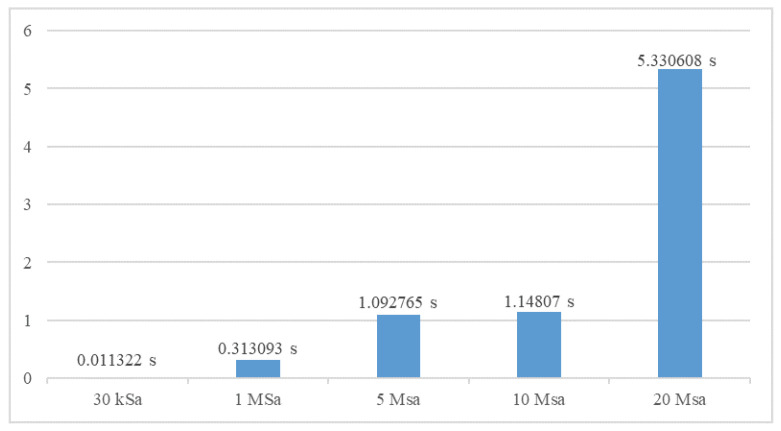
Pause time between measurements with threading functions.

Measuring current pulses one after another requires a time pause between each of the measurements. A single measurement usually generates large amount of data that must be stored before the next measurement starts. When operating with high sampling frequency time pause can become sufficiently large to limit number of recorded lightning events. Figure 16 shows measured pause time when recording with sampling frequencies in range from 30 kSa to 20 MSa. As indicated by time values in Figure 16, the observed time pause is considerable for sampling frequencies above 1 MSa. These results indicates that recording system is not capable to register lightning events that occur within 30 s (at 1 MSa) and 1050 s (at 20 MSa). To reduce large pause time at high sampling rates and optimize recording system performance, threading functions were used. Recording and data transfer processes are taking place in separate threading functions, which significantly reduces the pause between measurements, as shown in Figure 17. Using this approach considerable improvement is achieved and pause time is reduced on average by approximately 200 times. This enables recording successive lightning discharges at sampling rates up to 10 MSa within 1 s.

**Figure 18:**
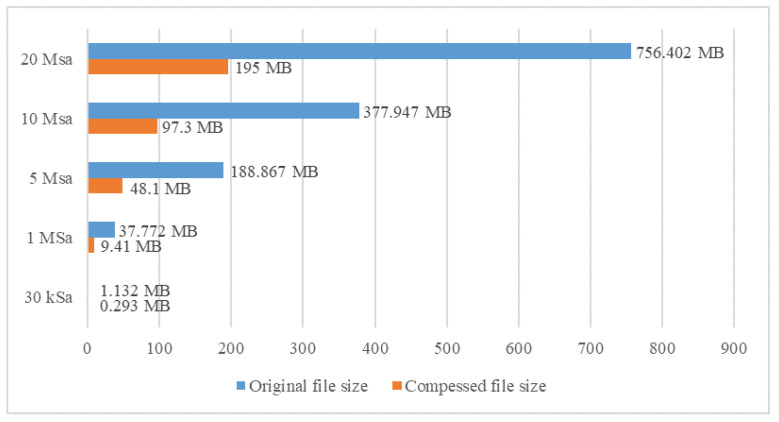
Size comparison of original file and compressed file.

**Figure 19:**
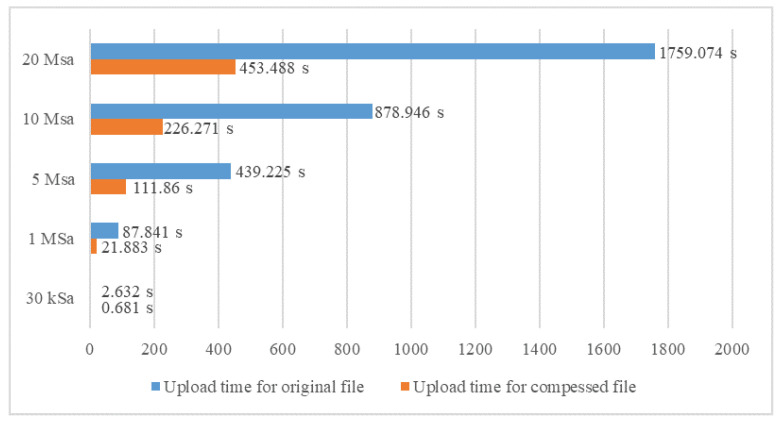
Upload time comparison for original file and compressed file.

Threading functions are also used to compress recorded data to reduce their size and time needed for upload to the Google Drive storage. This saves memory on both devices, the computer, and Google Drive. Also, it reduces mobile data traffic, which means faster and more reliable data upload. While performing these functions, the system can perform other measurements that are not related to the data being stored, converted, or uploaded in that particular moment. Figure 18 compares the sizes of saved data (in megabytes) when compressed versus when not compressed. The diagram in Figure 19 compares the uploading times when original data files are uploaded and when compressed data files are uploaded. The upload time is displayed in seconds. All data transfer and storage to Google Drive tests were successful.

If it is planned to install system in a remote location that is not easily accessible, it is crucial that it include an automated power-on option. During the testing period, numerous power outages were simulated. After each simulated power outage, the measuring system automatically restarted and was ready for the measurement. All previously chosen measurement parameters have also been restored. It is significant to note that during testing, there was no evidence of system component overheating.

## 5. The Cost and Power Consumption of Developed Transient Recording System

In this section, costs of developed system, power consumption and power supply methods were considered. The total cost of the developed system incorporates the price of measurement and communication equipment, the cost of power supply, and the cost of design and installation. The price of measurement and communication equipment is provided in Table 1. The overall consumption of measurement and communication equipment is presented in Table 2.

The power supply cost is presented in Table 3 and is formulated based on the proposals from local distributors of photovoltaic and electrical equipment [59,60,61,62]. The required battery Wh capacity and solar panel power are determined according to the assumed demand for continuous power supply for four consecutive cloudy days (4×24×30 W = 12×2×120 Ah). The installed power of photovoltaic modules is determined according to the assumed requirement for charging selected batteries during one day in the winter period (820 W × 4 h > 2880 Wh).

The installation of the system does not require special interventions on the structure. It is necessary to install solar panels, a cabinet enclosure for equipment, measurement probes (sensors), wiring, to configure the software, and to commission the system. Therefore, the cost of design and installation by trained personnel can amount to approximately 30% of the total project cost. Additional costs may include the operating cost of internet service (for remote locations) or advanced data analysis conducted by an external expert team. During the operation period, the battery in the autonomous power supply is the most critical component due to the limited number of charging/discharging cycles, with average operating life span of 5–7 years before replacement. Table 4 presents the overall cost of the developed system, including power supply and installation.

An estimated total cost of EUR 3895 is approximately a few times less expensive than the monitoring systems described in [13,21,22,23] without the power supply and additional costs of installation included. The cost of commercially available transient recorders from [24,25,26] are greater than approximately EUR 8000 for basic configuration.

Presented transient monitoring system is designed to be straightforward and user-friendly. The system is engineered with simplicity in mind, utilizing IIoT framework that allows for integration within existing power system infrastructure. Key considerations contributing to its ease of implementation include:

- The system is designed with a plug-and-play architecture, ensuring ease of installation. Components of the system are configured to be readily deployable, minimizing the need for complex setup procedures.
- To ensure integration with existing monitoring and control systems without requiring extensive modifications, the system is designed to be compatible with common power system interfaces and protocols.
- The real-time remote monitoring capability facilitates easy access to data.
- The system’s integration with Google Drive simplifies data management. The use of a widely accessible cloud platform ensures that recorded events are easily retrievable and shareable.
- The system is designed with low maintenance requirements, contributing to its ease of implementation. This feature minimizes downtime and operational disruptions.
- The cost-effective nature of the system makes it applicable to various power system facilities. Affordability of system components is a key factor in promoting widespread implementation.

In summary, the proposed transient monitoring system is engineered to be user-friendly, compatible with the existing power system infrastructure, and easily deployable. These features aim to facilitate a smooth integration process without encountering significant implementation challenges.

## 6. Conclusions

A significant amount of data must be gathered in order to improve evaluations of power system performance. Installing measurement equipment on the site and keeping an eye on transient occurrences in the power network is therefore very important. Due to the site-specific environment and transient phenomena that need to be captured, the system’s design is unique. High voltage measuring equipment is typically expensive and massive. The system presented in this work is IIoT solution-based, low cost, low consumption, compact to install, and capable of capturing power system transients.

The main advantages of this system are that it enables a general cost reduction, provides a compact system design with great performance, and uses less power. The system was continuously tested for three months. The tests that were run included: recording the currents of both polarities the positive and negative, data transfer and storage, and the auto on setting. The tests yield the following outcomes:

1. All generated lightning current shapes (standard waveform 8/20 µs) were successfully recorded;
2. The storing of files on Google Drive and all file transfers were successful, without any fail;
3. The measuring system immediately restarted and was prepared for the measurement after each simulated power interruption. This means that the auto-on option was successfully implemented;
4. It is important to emphasize that there was no trace of system component overheating throughout testing.

It can be concluded that the system’s developed functions have all been successfully implemented. Due to the significant practical value of proposed solutions in industrial applications, research and testing into their usage in high voltage monitoring systems must be conducted. Therefore, in next phase of this research, the proposed transient recording system will be installed at a 110 kV transmission line tower.

In the future, it will be necessary to install as many devices as possible that are cheap and compact, in order to collect more and more data to manage the power system better and predict the behavior of the power system due to certain events. Moreover, the developed system provides an opportunity for large dataset recording and analyzing using advanced techniques.

## Figures and Tables

**Table 1:** Costs of developed monitoring system.

System Component	Cost (EUR)
Rogowski coil with integrator	120
Voltage attenuator	20
Data acquisition unit	1300
Raspberry Pi 4B model	100
USB 3G Dongle and mobile data transfer	20
GPS/GNSS module and external antenna	50
Google Drive Storage 15 GB	FREE
Operating system—Raspberry Pi OS	FREE
Python—for software development	FREE
**Total:**	**1610**

**Table 2:** Power consumption of developed monitoring system.

System Component	Max. Power (W)
Voltage attenuator	2.0
Data acquisition Unit	10.0
Raspberry Pi 4B model	15.0
USB 3G Dongle and mobile data transfer	3.5
GPS/GNSS module and external antenna	0.1
**Total:**	**30.6**

**Table 3:** Specification of autonomous power supply with prices.

Component Description	Quantity	Unit Price (EUR)	Total (EUR)
Battery SOLE12V, 120 Ah, 12 V	2	154	308
Solar panel, 410 W, Amerisolar, HCAS24V	2	136	272
MPPT regulator, 12 V, 60 A	1	190	190
Supporting structure	1	131	131
Wiring	1	54	54
Enclosure	1	130	130
		**Total:**	**1085**

**Table 4:** The overall system cost.

Description	Price (EUR)
Measurement and communication equipment	1610
Autonomous power supply	1085
Installation and design	1200
**Total:**	**3895**

## Data Availability

The data that support the findings of this study are available from the corresponding author upon reasonable request.

## Abbreviations

The following abbreviations are used in this manuscript:

ADC	Analogue to Digital Converter
DAQ	Data Acquisition
EMI	Electromagnetic Interference
IIoT	Industrial Internet of Things
LLS	Lightning Location System
FPGA	Field Programmable Gate Arrays
GNSS	Global Navigation Satellite System
GPS	Global Positioning System
PLC	Programmable Logic Controller
PPS	pulse per second
SCADA	Supervisory Control and Data Acquisition
SIM	Subscriber Identification Module
